# Low composite functional movement screen score associated with decline of gait stability in young adults

**DOI:** 10.7717/peerj.11356

**Published:** 2021-04-30

**Authors:** Myeounggon Lee, Changhong Youm, Byungjoo Noh, Hwayoung Park

**Affiliations:** 1 Biomechanics Laboratory, Dong-A University, Busan, South Korea; 2 Health Care & Science, Dong-A University, Busan, South Korea; 3 Department of Kinesiology, Jeju National University, Jeju-si, South Korea

**Keywords:** Gait, Inertial measurement unit, Functional movement screen, Dynamic stability, Walking speed, Gait variability

## Abstract

**Background:**

The functional movement screen (FMS)^TM^ is a screening tool used to evaluate fundamental motor function. A score of 14 for the composite total FMS score (TFMS) is generally used as the cut-off point (≤14/21). In addition, gait analysis is used to evaluate fundamental motor function in humans. Thus, evaluating the fundamental motor function using the FMS^TM^ test and gait analysis at various speeds can provide further understanding of any decline in gait stability. In this study, we aimed to investigate the association between gait ability and fundamental movement patterns in young adults according to the cut-off point.

**Methods:**

A total of 439 participants (male: 203, female: 236) successfully completed the FMS test and a 1 min treadmill test; the participants were classified into two groups: low TFMS (≤14) and high TFMS (>14).

**Results:**

The low TFMS group exhibited slower and shortened walking patterns and worsen gait variability than the high TFMS group. The coefficient of variance (CV) for the double support phase at a faster speed (male) and the stride length at a slower speed (female) were classifiers between the two groups. In addition, the low TFMS group demonstrated insufficient gait adaptation at the preferred and faster speeds based on the CV of the double support phase and gait asymmetry. Lower TFMS is associated with a decline in gait ability. Therefore, participants with a lower TFMS and poor gait ability may require intervention programs to prevent risk of future injury and to enhance motor function.

## Introduction

Today, individuals attempt to enhance their strength and health through physical activity and exercise, and numerous athletes and individuals perform high-intensity physical activities or exercise to accomplish this goal (Cook et al., 2014; Scudamore et al., 2019). However, if individuals that regularly participate in physical activities do not address their weaknesses, they will compensate using inefficient fundamental movements that can lead to decline in performance as well as an increase in potential risk for injuries, and their physical function can gradually deteriorate (Cook et al., 2014; Scudamore et al., 2019; Letafatkar et al., 2014). Therefore, screening the fundamental movement function using a valid and reliable assessment method is important so that these weaknesses can be addressed.

The Functional Movement Screen (FMS)™ is a screening tool used to evaluate mobility, balance, and postural stability using seven fundamental movement patterns and three additional clearing tests (Cook et al., 2014; Scudamore et al., 2019; Cook, Burton & Hoogenboom, 2006a; Cook, Burton & Hoogenboom, 2006b). Acceptable results for the intra- and interclass reliability (coefficients of 0.869 and 0.843, respectively) of the total FMS score (TFMS), calculated using the sum of the seven movement scores (Scudamore et al., 2019; Moran et al., 2017; Trinidad-Fernandez, Gonzalez-Sanchez & Cuesta-Vargas, 2019; Bonazza et al., 2017; Moore et al., 2019), have been reported (Cuchna, Hoch & Hoch, 2016; Moran et al., 2017).

Numerous researchers and practitioners have used the FMS™ battery to identify risk for musculoskeletal injury over the past decade (Scudamore et al., 2019). Recent studies have considered a TFMS ≤ 14 out of 21 as a cut-off point to predict potential risk of musculoskeletal injury (Scudamore et al., 2019; Bonazza et al., 2017). However, several researchers have argued that a TFMS ≤ 14 may be inadequate to predict the future risk of injury because these studies did not consider personal characteristics, such as age, sex, sports, and injury types in athletes (Moran et al., 2017; Trinidad-Fernandez, Gonzalez-Sanchez & Cuesta-Vargas, 2019; Moore et al., 2019). Nevertheless, the TFMS cut-off score of 14 may be considered an appropriate threshold to identify poor balance in an active young male and female population, excluding athletes and military personnel (Scudamore et al., 2019). Thus, individuals with a TFMS ≤ 14 may show poorer performance in parameters such as stability, flexibility, and balance than those with a TFMS >14.

Gait is a fundamental motor skill for humans, providing forward movement and stability to the human body during walking (Perry & Davids, 1992). Gait analysis has been used to evaluate physical function, health status, and quality of life and is conducted for healthy individuals as well as for patients (McKay et al., 2017). For instance, slower and shortened walking patterns reflect weakness of the lower limbs (Herssens et al., 2018; Aboutorabi et al., 2016). Furthermore, gait variability (GV) (Almarwani et al., 2016; Rennie et al., 2018) and bilateral coordination (Plotnik, Giladi & Hausdorff, 2007; Nasirzade et al., 2017) are variables that can be used to evaluate dynamic stability during walking; higher values of GV and bilateral coordination indicate poor dynamic stability during walking. These patterns may worsen with aging and disease factors (Almarwani et al., 2016; Plotnik, Giladi & Hausdorff, 2007; Mirelman et al., 2015). The FMS test is designed to evaluate basic locomotor, manipulative, and stabilizing movements, and if participants with lower quality of fundamental movement functions undertake the test, they could exhibit poor stability, mobility, and dynamic balance (Cook et al., 2014; Scudamore et al., 2019). Thus, evaluating the gait ability with a TFMS and using a cut-off score of 14 may provide meaningful results to understand the fundamental motor function in young adults; however, few studies have evaluated the association between the TFMS and gait ability. Recently, an inertial measurement unit (IMU) system for gait analysis has exhibited validity and reliability in healthy individuals (Joo, Kim & Park, 2015; Kanzler et al., 2015; Mariani et al., 2010) and patients (Trojaniello et al., 2014; Trojaniello et al., 2015; Lee et al., 2018); thus, numerous researchers have suggested this alternative approach for gait analysis (Joo, Kim & Park, 2015; Kanzler et al., 2015; Mariani et al., 2010; Trojaniello et al., 2014; Lee et al., 2018; Cole et al., 2014). Several studies that used the IMU system have reported a significant decline in gait ability based on quantitative speed conditions (e.g.,  ±20% of the preferred walking speed of an individual), and gait tasks at these speed conditions have been validated in healthy young adults (Han et al., 2019), older adults (Noh et al., 2020; Lee et al., 2020a), and patients with Parkinson’s disease (Lee et al., 2020b). Thus, gait tasks at slower, faster, and self-preferred speeds using the IMU system may demonstrate specific characteristics in young adults, based on a TFMS cut-off score of 14 out of 21.The purpose of this study was to investigate the association between gait ability and fundamental movement patterns in young adults using a TFMS with a cut-off score of 14. We hypothesized that individuals with a TFMS of ≤14 (low TFMS group) would exhibit worse gait stability with slower and shortened patterns and worsen values of GV and bilateral coordination than individuals with a TFMS of >14 (high TFMS group). We also suspected that different characteristics would be observed based on sex. In addition, we expected that the low TFMS group would exhibit an insufficient gait adaptation ability with an increase in walking speed. Finally, we assumed that the TFMS would exhibit significant associations with gait variables as well as with physical characteristics, stress level, and physical activity (PA) levels.

**Table 1:** Demographic characteristics.

	All participants *N* = 439		Male *N* = 203		Female *N* = 236		Significance for groups
	Low TFMS (≤ 14) *N* = 196	High TFMS (>14) *N* = 243	Low TFMS (≤ 14) *N* = 83	High TFMS (>14) *N* = 120	Low TFMS (≤ 14) *N* = 113	High TFMS (>14) *N* = 123	
Age (years)	22.74 ± 2.28	22.93 ± 2.31	23.33 ± 2.45	23.46 ± 2.38	22.31 ± 2.05	22.42 ± 2.12	N/S
Height (cm)	167.27 ± 8.28	167.65 ± 8.61	175.02 ± 5.02	174.19 ± 6.04	161.58 ± 4.85	161.26 ± 5.31	N/S
Body weight (kg)	64.88 ± 12.35	64.01 ± 12.93	73.45 ± 10.49	72.08 ± 10.95	58.58 ± 9.52	56.14 ± 9.37	C
BMI (kg/m^2^)	23.08 ± 3.45	22.63 ± 3.38	23.98 ± 3.23	23.73 ± 3.14	22.43 ± 3.47	21.57 ± 3.28	N/S
Percent Body Fat (%)	26.82 ± 8.17	24.07 ± 7.72	20.79 ± 6.79	18.59 ± 5.65	31.28 ± 5.93	29.42 ± 5.36	A, B, C
TFMS (score)	12.64 ± 1.51	16.21 ± 1.18	12.49 ± 1.53	16.29 ± 1.23	12.75 ± 1.49	16.13 ± 1.13	A, B, C
SRI-MF (score)	31.42 ± 17.85	25.30 ± 17.83	29.74 ± 18.91	21.04 ± 16.36	32.65 ± 17.00	29.45 ± 18.28	A, B
Vigorous PA (METs)	660.00 ± 1,144.35	823.87 ± 1,466.04	1,087.71 ± 1,326.38	1,146.00 ± 1,667.97	345.49 ± 869.81	509.59 ± 1,161.00	N/S
Moderate PA (METs)	256.63 ± 412.97	433.50 ± 721.93	322.89 ± 521.86	569.50 ± 797.37	207.96 ± 303.14	300.81 ± 614.57	A, B
Walking PA (METs)	946.06 ± 964.90	933.77 ± 984.88	891.00 ± 658.35	1,026.30 ± 936.29	986.50 ± 1,140.08	849.41 ± 1,026.36	N/S
Total PA (METs)	1,862.48 ± 1,912.03	2,194.13 ± 2,366.39	2,301.60 ± 1,848.46	2,741.80 ± 2,547.07	1,539.95 ± 1,901.49	1,659.82 ± 2,048.63	N/S

**Notes.**

Mean ± standard deviation.

BMI Body mass index METs Metabolic equivalents TFMS Total Functional Movement screen score, SRI-MF Stress response index modified from PA Physical activity

(A) Significant difference between all participants in the low TFMS and high TFMS groups; (B) Significant difference between male participants of the low TFMS and high TFMS groups; (C) a significant difference between female participants of the low TFMS and high TFMS groups; *p* < 0.05; N/S indicates no significance.

## Materials & Methods

### Study population

**Figure 1:**
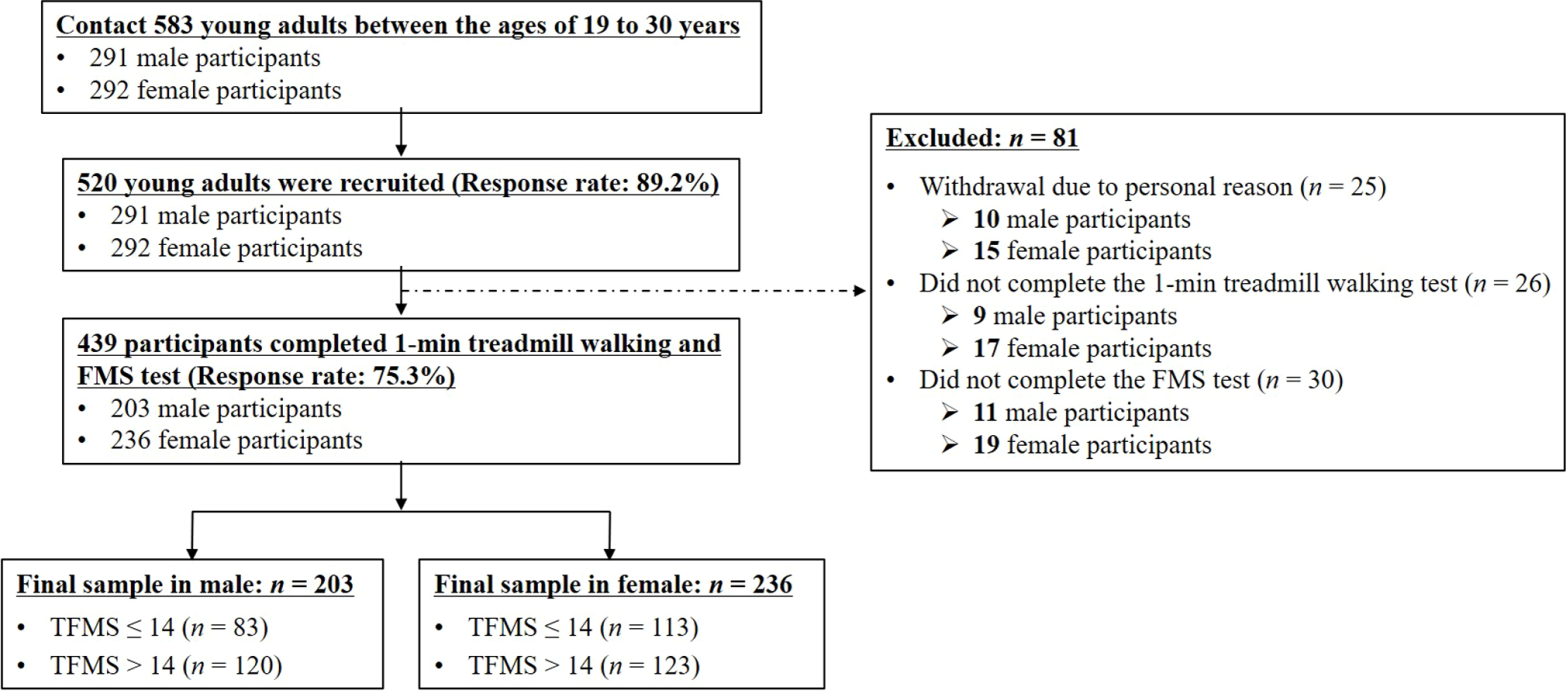
Flowchart for recruitment.

Participants were recruited as part of a community-wide survey in Busan, a metropolitan city, from February to December 2018. We contacted 583 young adults 19 to 30 years of age living in the community, and recruited 520 of them for this study (response rate: 89.2%). None of the participants had a history of musculoskeletal or neurological problems that affected gait, and all participants could walk without support. We excluded 81 participants from the study owing to personal reasons (*n* = 25), non-completion of the 1 min treadmill walking test (*n* = 26) and FMS test (*n* = 30). A total of 439 participants (male: 203, female: 236) successfully completed the test trials (response rate: 75.3%) (Table 1). A score of 14 for the TFMS was considered the threshold to predict potential injury risks in athletes or military populations (Scudamore et al., 2019; Bonazza et al., 2017; Mirelman et al., 2015); and the TFMS may be useful in a broader population of adults, with a valid result (Cronbach’s alpha: 0.64; Koehle et al., 2016 ), and the score of 14 may be an appropriate threshold for identifying poor balance in an active population of young male and female subjects (Scudamore et al., 2019). The mean of TFMS was reported to be approximately 14 for the general population in large samples (622 and 1,133 participants), excluding athletes and military personnel (Koehle et al., 2016; Perry & Koehle, 2013). Therefore, despite conflicting reports regarding the association of a low TFMS ( ≤ 14), with the risk of future injury (Scudamore et al., 2019; Moran et al., 2017; Trinidad-Fernandez, Gonzalez-Sanchez & Cuesta-Vargas, 2019; Bonazza et al., 2017; Moore et al., 2019), we considered a TFMS of 14 as a threshold in this study. We classified the participants according to their TFMS into the low TFMS (TFMS ≤ 14 points) and high TFMS (TFMS >14 points) groups (Koehle et al., 2016; Perry & Koehle, 2013; Scudamore et al., 2019); see Table 1 and Fig. 1). All participants read and signed an informed consent form that was approved by the Institutional Review Board of Dong-A University (IRB number: 2-1040709-AB-N-01-201805-HR-010-02). Moreover, this study was performed in accordance with the Declaration of Helsinki and the ethical guidelines for human subjects of the Institutional Review Board of Dong-A University.

### Evaluating the FMS

The FMS™ is a battery composed of seven fundamental movement patterns that are used to evaluate dysfunctional, asymmetrical, and painful movements contributing to future injuries (Cook, Burton & Hoogenboom, 2006a; Cook, Burton & Hoogenboom, 2006b). It includes seven fundamental movement patterns (deep squat, hurdle step, in-line lunge, shoulder mobility, active straight leg raise, trunk stability push-up, and rotary stability) that are used to evaluate the balance of mobility and stability (Cook, Burton & Hoogenboom, 2006a; Cook, Burton & Hoogenboom, 2006b) using an evaluation kit (FMS kits, Functional movement system Inc, USA). Each movement was scored on a quartile scale, which included pain (score: 0), incomplete movement (score: 1), movement with a compensation pattern (score: 2), and correct movement without any compensation (score: 3). Specific comments were noted to specify why a score of three was not obtained. The scores of the seven movements were added to obtain the TFMS, and the maximum TFMS was 21; a higher TFMS reflects better movement patterns (Moore et al., 2019). Our researchers who evaluated the FMS test were trained to measure functional movement and check measurement time, and each movement was conducted two to three times to observe movement patterns from different planes (Cook et al., 2014; Cook, Burton & Hoogenboom, 2006a; Cook, Burton & Hoogenboom, 2006b). We evaluated and recorded the scores for both the left and right sides, and the lower score was used as the score for each movement task (Cook et al., 2014; Cook, Burton & Hoogenboom, 2006a; Cook, Burton & Hoogenboom, 2006b).

### Instrumentation

Shoe-type IMU systems (DynaStab™, JEIOS, South Korea), which included shoe-type data loggers (Smart Balance SB-1^®^, JEIOS, South Korea) and a data acquisition system, were utilized in this study. The shoe-type data logger included an IMU sensor (IMU–3000™, InvenSense, USA) on the outsoles of both shoes that could measure the triaxial acceleration (up to ±6 g) and triaxial angular velocities (up to ±500° s^−1^) along the three orthogonal axes. The data were transmitted wirelessly to a data acquisition system via Bluetooth^®^(Joo, Kim & Park, 2015; Lee et al., 2018; Kim et al., 2016). The shoe sizes were adapted to fit the tested individuals, and a range of shoe sizes was available (from 225 to 280 mm).

### Test procedures

All test procedures, such as the measurement of the demographic characteristics, questionnaires, FMS test, and gait tasks, were completed in a single day. The biometric data, including body height, weight, and body fat percentage (Inbody 270, InBody Co, South Korea), were recorded before the FMS and treadmill walking test. Then, all participants answered questionnaires to assess their physical activity (PA) and stress response. PA was evaluated using the international PA questionnaire–short form (IPAQ–SF), which included seven items that pertained to the self-reported PA of the participants, such as high, moderate, and walking PA. Based on these questionnaires, we calculated the metabolic equivalents (METs/week) (Oyeyemi et al., 2014). Perry & Koehle, (2013) reported the TFMS was associated with the BMI and PA levels, but they suggested considering additional variables that may influence the TFMS. Thus, we considered additional variable related to the quality of life, and we added a questionnaire to evaluate stress level. The stress response was assessed using the modified stress response inventory (SRI-MF), which included 22 questions. Each question was scored on the Likert scale and included “not at all,” “somewhat,” “moderately,” “very much,” and “absolutely” (Choi, Kang & Woo, 2006). These 22 questions were categorized into three simplified stress factors—somatization, depression, and anger. Cronbach’s alphas for the SRI (Koh et al., 2001) were indicated by somatization (0.89), depression (0.88), and anger (0.87) (Choi, Kang & Woo, 2006). A higher SRI-MF total score indicated severe stress levels.

**Figure 2:**
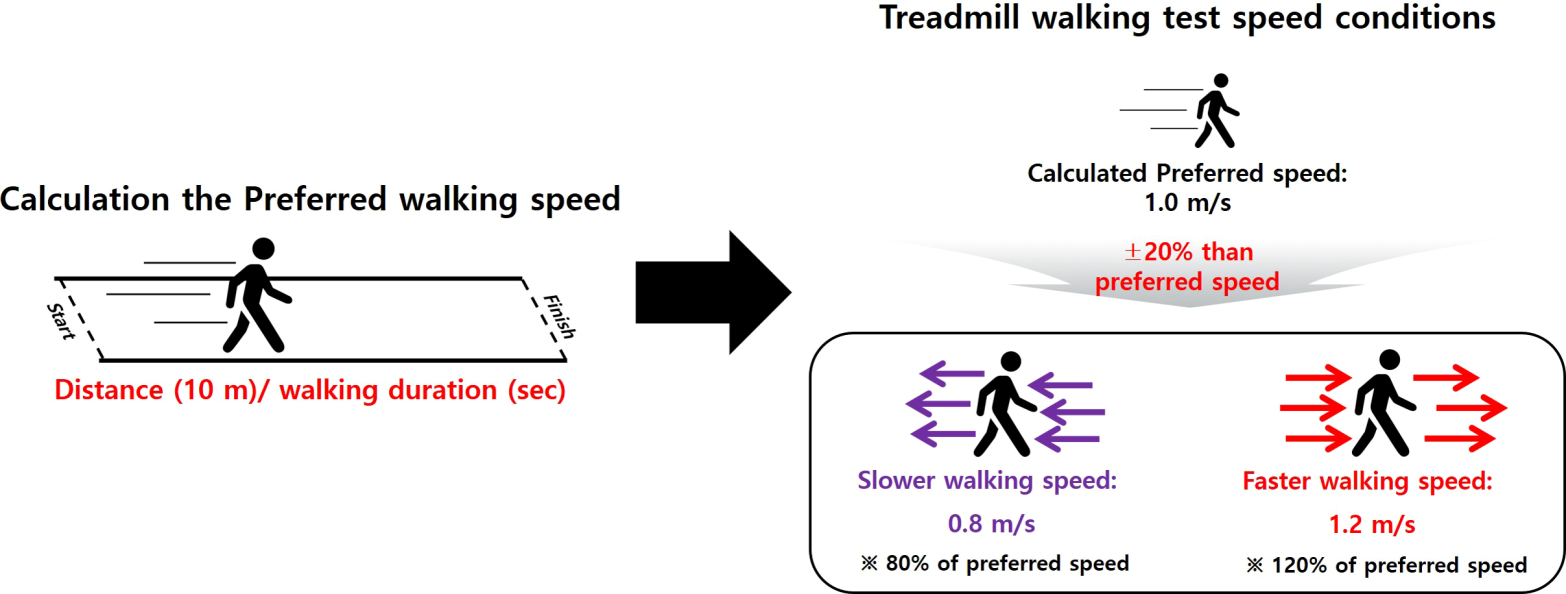
Walking speed definitions. Slower (80% of the preferred) and faster (120% of the preferred) speeds, calculated based on the participant’s preferred speed.

After completing the demographic characteristic measurements and questionnaires, all participants performed the FMS test according to the FMS guideline (Cook et al., 2014; Cook, Burton & Hoogenboom, 2006a; Cook, Burton & Hoogenboom, 2006b). In addition, all participants performed overground walking tests on a straight 10 m walkway before the treadmill test to calculate their self-preferred walking speed (distance/walking duration). Slower (80% of preferred speed) and faster (120% of preferred speed) speeds were calculated relative to the preferred speed (Noh et al., 2020; Lee et al., 2020a; Lee et al., 2020b). For instance, if the measured preferred speed was 1.0 m/s, then the slower and faster speeds were 0.8 m/s and 1.2 m/s, respectively (Fig. 2). Subsequently, the participants practiced their respective self-preferred speed, along with the slower and faster speeds, on the treadmill (HK–365, Healthkeeper, South Korea) until they felt comfortable (approximately 1 to 3 min). After practicing for speed adaptation, the participants rested for approximately 5 min. During the treadmill walking test, the participants were asked to walk while maintaining a stable walking pattern for each speed condition on the treadmill for approximately 30–60 s at the onset of treadmill walking. An operator collected the treadmill walking data at 100 Hz (1 min periods) when a participant attained a stable walking pattern (Lee et al., 2020c). The treadmill walking procedure followed this sequence: (1) self-preferred speed; (2) faster speed; (3) slower speed. The participants rested approximately 1 to 2 min between the treadmill walking trials to reduce muscle fatigue.

### Data analyses

The treadmill walking data were filtered with a second-order Butterworth low-pass filter with a cut-off frequency of 10 Hz (Joo, Kim & Park, 2015; Lee et al., 2018; Kim et al., 2016). A heel strike event is defined as an event where the linear acceleration on the anteroposterior axis reaches its maximum positive (+) value, and a toe off event is defined as one where the linear acceleration on the vertical axis reaches its maximum positive (+) value during the gait cycle (Lee et al., 2018; Kim et al., 2016). Spatiotemporal parameters were calculated as follows: walking speed, stride length, step length, single support, double support, stance phase, cadence, stride time, and step time. For GV, the values of percentage coefficient of variance (CV) (standard deviation/mean) ×100) were also calculated for all the spatiotemporal parameters . To calculate bilateral coordination, the gait asymmetry (GA) and phase coordinate index (PCI) were calculated based on a previous study, and the increased GA and PCI values indicated reduced bilateral coordination, reflecting reduced gait stability (Plotnik, Giladi & Hausdorff, 2007; Plotnik et al., 2013).

### Statistical analyses

All statistical analyses were performed using a commercially available statistics software program for Windows (version 21.0, SPSS Inc. Chicago, IL). An independent sample *t*-test was performed to compare the low TFMS and high TFMS groups (*p* <0.05). A one-way repeated measures analysis of variance with *Bonferroni* correction (*p*-value: 0.05/3, 0.0167) was used to compare the slower, preferred, and faster speed conditions. In addition, a binary logistic regression analysis using the forward and backward stepwise method was performed to determine the classifiers between the low TFMS and high TFMS groups according to all participants and sexes based on the gait-related variables. Prior to the logistic regression analysis, a univariable logistic analysis was conducted to minimize the multicollinearity problems. Then, a multivariable stepwise binary logistic regression analysis was performed. The model was adjusted for the body mass index (BMI) and percent body fat.

Furthermore, after determining the relationships between the TFMS and functional and gait characteristics, Pearson’s product-moment correlation analysis was performed to compare the TFMS and functional characteristics, such as PA (IPAQ–SF) and stress response (SRI-MF), as well as the gait-related variables for all participants and sexes. The statistical significance was set to 0.05.

## Results

### Group differences: low TFMS vs. high TFMS groups

Among all participants, the low TFMS group exhibited significantly higher GV parameters (such as the CVs of stride length, step length, single support phase, stance phase, stride time, and step time) at faster speeds, as well as the PCI at slower speeds than the high TFMS group (Table 2). For male participants, the GV parameters at faster speeds , such as the CVs of stride length, single support phase, double support phase, stance phase, stride time, and step time, as well as the PCI at slower speeds, were significantly higher in the low TFMS group than in the high TFMS group (Table 3). For female participants, the low TFMS group exhibited significantly higher GV parameters at the slower and faster speeds for the CVs of stride length and stride time than the high TFMS group (Table 4). The low TFMS group exhibited significantly lower stride and step length at all speed conditions than the high TFMS group for all participants and in the case of male participants. No significant differences in the spatiotemporal parameters were observed for the female participants.

**Table 2:** Group and speed differences of the gait variables for all participants.

Variables	**Slower speed**		**Preferred speed**		**Faster speed**		Significance for groups	Significance for speeds	
	Low TFMS (≤ 14)	**High TFMS** (>14)	**Low TFMS** (≤ 14)	High TFMS (>14)	Low TFMS (≤ 14)	High TFMS (>14)		Low TFMS (≤ 14)	**High TFMS** (>14)
**Spatiotemporal parameters**									
Walking speed (m/s)	0.90 ± 0.13	0.93 ± 0.13	1.23 ± 0.17	1.16 ± 0.16	1.35 ± 0.21	1.39 ± 0.19	N/S	a, b, c	a, b, c
Stride length (m)	1.18 ± 0.14	1.22 ± 0.13	1.29 ± 0.14	1.32 ± 0.14	1.43 ± 0.16	1.48 ± 0.16	A, B, C	a, b, c	a, b, c
Step length (m)	0.59 ± 0.07	0.61 ± 0.07	0.64 ± 0.07	0.66 ± 0.07	0.72 ± 0.08	0.74 ± 0.08	A, B, C	a, b, c	a, b, c
Single support phase (%)	37.68 ± 1.41	37.61 ± 1.36	39.05 ± 1.36	39.04 ± 1.26	40.29 ± 1.32	40.33 ± 1.27	N/S	a, b, c	a, b, c
Double support phase (%)	24.79 ± 2.47	24.86 ± 2.66	21.90 ± 2.47	21.91 ± 2.52	19.41 ± 2.49	19.29 ± 2.54	N/S	a, b, c	a, b, c
Stance phase (%)	62.48 ± 1.34	62.47 ± 1.52	60.96 ± 1.26	60.95 ± 1.39	59.70 ± 1.30	59.62 ± 1.38	N/S	a, b, c	a, b, c
Cadence (steps/min)	91.19 ± 7.57	90.75 ± 7.14	104.43 ± 7.54	104.25 ± 7.31	112.36 ± 7.91	112.21 ± 7.08	N/S	a, b, c	a, b, c
Stride time (s)	1.32 ± 0.11	1.32 ± 0.11	1.15 ± 0.09	1.15 ± 0.08	1.07 ± 0.08	1.07 ± 0.07	N/S	a, b, c	a, b, c
Step time (s)	0.66 ± 0.06	0.66 ± 0.05	0.57 ± 0.04	0.58 ± 0.04	0.53 ± 0.04	0.53 ± 0.04	N/S	a, b, c	a, b, c
**Gait variability parameters**									a, b, c
CV of stride length (%)	2.66 ± 0.89	2.55 ± 0.91	1.86 ± 0.57	1.79 ± 0.60	1.57 ± 0.47	1.42 ± 0.45	C	a, b, c	a, b, c
CV of step length (m)	1.76 ± 0.68	1.72 ± 0.64	1.08 ± 0.37	1.04 ± 0.40	0.85 ± 0.30	0.77 ± 0.28	C	a, b, c	a, b, c
CV of single support phase (%)	5.93 ± 2.30	5.75 ± 2.30	3.62 ± 1.32	3.44 ± 1.29	2.82 ± 1.02	2.57 ± 0.83	C	a, b, c	a, b, c
CV of double support phase (%)	10.03 ± 4.19	9.56 ± 3.83	7.14 ± 2.15	7.09 ± 2.20	6.93 ± 2.13	6.59 ± 2.15	N/S	a, b	a, b, c
CV of stance phase (%)	4.33 ± 1.64	4.10 ± 1.48	2.72 ± 0.89	2.59 ± 0.86	2.12 ± 0.68	1.95 ± 0.60	C	a, b, c	a, b, c
CV of stride time (s)	2.66 ± 0.89	2.55 ± 0.91	1.86 ± 0.57	1.79 ± 0.60	1.57 ± 0.47	1.42 ± 0.45	C	a, b, c	a, b, c
CV of step time (s)	2.65 ± 0.89	2.57 ± 0.87	1.86 ± 0.56	1.80 ± 0.61	1.58 ± 0.49	1.44 ± 0.51	C	a, b, c	a, b, c
**Bilateral coordination**									a, b, c
GA (%)	2.36 ± 2.19	2.26 ± 1.97	1.75 ± 1.40	1.71 ± 1.37	1.61 ± 1.24	1.48 ± 1.16	N/S	a, b	a, b, c
PCI (%)	4.54 ± 1.48	4.19 ± 1.39	3.51 ± 1.02	3.33 ± 1.11	3.08 ± 0.86	2.92 ± 0.93	A	a, b, c	a, b, c

**Notes.**

CV coefficient of variance GA Gait asymmetry PCI Phase coordinate index TFMS Total functional movement screen score

Group differences between low TFMS and high TFMS for slower (A), preferred (B), and faster (C) speeds; *p* < 0.05; Speed differences within the slower vs. preferred (a), slower vs. faster (b), preferred vs. faster (c) speeds; *p* < 0.0167 (0.05/3); N/S indicates no significance.

**Table 3:** Group and speed differences of the gait variables for male participants.

Variables	**Slower speed**		**Preferred speed**		**Faster speed**		Significance for groups	Significance for speeds	
	Low TFMS (≤ 14)	High TFMS (>14)	**Low TFMS** (≤ 14)	**High TFMS** (>14)	**Low TFMS** (≤ 14)	High TFMS (>14)		**Low TFMS** **(≤ 14)**	**High TFMS** (>14)
**Spatiotemporal parameters**									
Walking speed (m/s)	0.94 ± 0.12	0.96 ± 0.11	1.18 ± 0.16	1.20 ± 0.14	1.42 ± 0.19	1.44 ± 0.17	N/S	a, b, c	a, b, c
Stride length (m)	1.23 ± 0.13	1.27 ± 0.11	1.34 ± 0.14	1.38 ± 0.12	1.50 ± 0.15	1.55 ± 0.14	A, B, C	a, b, c	a, b, c
Step length (m)	0.62 ± 0.06	0.63 ± 0.06	0.67 ± 0.07	0.69 ± 0.06	0.75 ± 0.07	0.77 ± 0.07	A, B, C	a, b, c	a, b, c
Single support phase (%)	38.01 ± 1.31	37.75 ± 1.34	39.36 ± 1.21	39.06 ± 1.28	40.55 ± 1.16	40.38 ± 1.29	N/S	a, b, c	a, b, c
Double support phase (%)	24.18 ± 2.13	24.53 ± 2.56	21.43 ± 2.21	21.79 ± 2.45	19.08 ± 2.15	19.23 ± 2.51	N/S	a, b, c	a, b, c
Stance phase (%)	62.18 ± 1.15	62.28 ± 1.47	60.78 ± 1.17	60.85 ± 1.33	59.64 ± 1.16	59.61 ± 1.32	N/S	a, b, c	a, b, c
Cadence (steps/min)	91.54 ± 7.16	90.62 ± 6.86	105.10 ± 6.61	103.73 ± 6.91	112.67 ± 7.02	111.52 ± 6.68	N/S	a, b, c	a, b, c
Stride time (s)	1.31 ± 0.11	1.32 ± 0.10	1.14 ± 0.07	1.16 ± 0.08	1.06 ± 0.07	1.07 ± 0.07	N/S	a, b, c	a, b, c
Step time (s)	0.66 ± 0.05	0.66 ± 0.05	0.57 ± 0.04	0.58 ± 0.04	0.53 ± 0.03	0.54 ± 0.03	N/S	a, b, c	a, b, c
**Gait variability parameters**									
CV of stride length (%)	2.68 ± 0.83	2.69 ± 1.05	1.77 ± 0.56	1.71 ± 0.60	1.54 ± 0.54	1.38 ± 0.43	C	a, b, c	a, b, c
CV of step length (m)	1.77 ± 0.68	1.78 ± 0.69	1.03 ± 0.35	1.00 ± 0.39	0.83 ± 0.33	0.75 ± 0.25	N/S	a, b, c	a, b, c
CV of single support phase (%)	5.78 ± 2.32	5.76 ± 2.30	3.36 ± 1.08	3.30 ± 1.17	2.73 ± 1.10	2.45 ± 0.67	C	a, b, c	a, b, c
CV of double support phase (%)	9.95 ± 4.50	9.47 ± 3.81	7.09 ± 2.25	6.85 ± 2.24	7.06 ± 2.41	6.29 ± 1.87	C	a, b	a, b, c
CV of stance phase (%)	4.31 ± 1.68	4.20 ± 1.52	2.64 ± 0.89	2.53 ± 0.87	2.09 ± 0.78	1.90 ± 0.57	C	a, b, c	a, b, c
CV of stride time (s)	2.68 ± 0.83	2.69 ± 1.05	1.77 ± 0.56	1.71 ± 0.60	1.54 ± 0.53	1.38 ± 0.43	C	a, b, c	a, b, c
CV of step time (s)	2.67 ± 0.87	2.67 ± 0.98	1.79 ± 0.54	1.71 ± 0.62	1.53 ± 0.53	1.39 ± 0.43	C	a, b, c	a, b, c
**Bilateral coordination**									
GA (%)	2.30 ± 2.34	2.39 ± 2.00	1.88 ± 1.30	1.74 ± 1.41	1.70 ± 1.32	1.38 ± 1.13	N/S	b	a, b, c
PCI (%)	4.60 ± 1.65	4.14 ± 1.49	3.44 ± 1.08	3.17 ± 1.00	3.02 ± 0.87	2.84 ± 0.89	A	a, b, c	a, b, c

**Notes.**

CV coefficient of variance GA Gait asymmetry PCI Phase coordinate index; TFMS: Total functional movement screen score

Group differences between low TFMS and high TFMS for slower (A), preferred (B), and faster (C) speeds; *p* < 0.05; Speed differences within the slower vs. preferred (a), slower vs. faster (b), preferred vs. faster (c) speeds; *p* < 0.0167 (0.05/3); N/S indicates no significance.

**Table 4:** Group and speed differences of gait variables for female participants.

**Variables**	**Slower speed**		**Preferred speed**		**Faster speed**		Significance for groups	Significance for speeds	
	**Low TFMS** (≤ 14)	**High TFMS** (>14)	**Low TFMS** (≤ 14)	**High TFMS** (>14)	**Low TFMS** (≤ 14)	**High TFMS** (>14)		Low TFMS **(≤ 14)**	**High TFMS** (>14)
**Spatiotemporal parameters**									
Walking speed (m/s)	0.87 ± 0.13	0.89 ± 0.13	1.09 ± 0.17	1.11 ± 0.16	1.31 ± 0.20	1.34 ± 0.20	N/S	a, b, c	a, b, c
Stride length (m)	1.14 ± 0.13	1.17 ± 0.13	1.25 ± 0.13	1.26 ± 0.14	1.39 ± 0.14	1.41 ± 0.16	N/S	a, b, c	a, b, c
Step length (m)	0.57 ± 0.07	0.58 ± 0.06	0.62 ± 0.07	0.63 ± 0.07	0.69 ± 0.07	0.70 ± 0.08	N/S	a, b, c	a, b, c
Single support phase (%)	37.44 ± 1.44	37.47 ± 1.37	38.83 ± 1.43	39.02 ± 1.26	40.10 ± 1.41	40.28 ± 1.25	N/S	a, b, c	a, b, c
Double support phase (%)	25.25 ± 2.61	25.18 ± 2.73	22.27 ± 2.60	22.03 ± 2.58	19.65 ± 2.70	19.35 ± 2.59	N/S	a, b, c	a, b, c
Stance phase (%)	62.69 ± 1.43	62.65 ± 1.55	61.09 ± 1.30	61.05 ± 1.45	59.75 ± 1.40	59.63 ± 1.45	N/S	a, b, c	a, b, c
Cadence (steps/min)	90.94 ± 7.88	90.89 ± 7.44	103.95 ± 8.15	104.76 ± 7.67	112.13 ± 8.52	112.88 ± 7.41	N/S	a, b, c	a, b, c
Stride time (s)	1.32 ± 0.12	1.32 ± 0.11	1.16 ± 0.09	1.14 ± 0.10	1.07 ± 0.08	1.06 ± 0.07	N/S	a, b, c	a, b, c
Step time (s)	0.66 ± 0.06	0.66 ± 0.06	0.58 ± 0.05	0.57 ± 0.04	0.54 ± 0.04	0.53 ± 0.04	N/S	a, b, c	a, b, c
**Gait variability parameters**									
CV of stride length (%)	2.65 ± 0.93	2.42 ± 0.73	1.92 ± 0.57	1.87 ± 0.58	1.59 ± 0.42	1.45 ± 0.48	A, C	a, b, c	a, b, c
CV of step length (m)	1.76 ± 0.68	1.65 ± 0.59	1.12 ± 0.38	1.09 ± 0.40	0.87 ± 0.28	0.79 ± 0.31	N/S	a, b, c	a, b, c
CV of single support phase (%)	6.03 ± 2.29	5.74 ± 2.31	3.81 ± 1.44	3.58 ± 1.38	2.89 ± 0.97	2.66 ± 0.95	N/S	a, b, c	a, b, c
CV of double support phase (%)	10.08 ± 3.98	9.64 ± 3.86	7.17 ± 2.09	7.32 ± 2.13	6.84 ± 1.91	6.88 ± 2.37	N/S	a, b	a, b
CV of stance phase (%)	4.35 ± 1.61	4.00 ± 1.44	2.78 ± 0.90	2.65 ± 0.85	2.14 ± 0.61	2.00 ± 0.63	N/S	a, b, c	a, b, c
CV of stride time (s)	2.65 ± 0.93	2.42 ± 0.73	1.92 ± 0.57	1.87 ± 0.58	1.59 ± 0.42	1.45 ± 0.48	A, C	a, b, c	a, b, c
CV of step time (s)	2.64 ± 0.92	2.48 ± 0.75	1.91 ± 0.57	1.88 ± 0.60	1.61 ± 0.46	1.49 ± 0.58	N/S	a, b, c	a, b, c
**Bilateral coordination**									
GA (%)	2.41 ± 2.08	2.13 ± 1.95	1.65 ± 1.47	1.69 ± 1.34	1.54 ± 1.17	1.58 ± 1.19	N/S	a, b	a, b
PCI (%)	4.50 ± 1.35	4.23 ± 1.55	3.57 ± 0.98	3.49 ± 1.19	3.13 ± 0.86	2.99 ± 0.96	N/S	a, b, c	a, b, c

**Notes.**

CV coefficient of variance GA Gait asymmetry PCI Phase coordinate index TFMS Total functional movement screen score

Group differences between low TFMS and high TFMS for slower (A), preferred (B), and faster (C) speeds; *p* < 0.05; Speed differences within the slower vs. preferred (a), slower vs. faster (b), preferred vs. faster (c) speeds; *p* < 0.0167 (0.05/3); N/S indicates no significance.

### Speed differences: gait-related variables at slower, preferred, and faster speeds

The low TFMS group exhibited a significant difference at the slower, preferred, and faster speeds for all spatiotemporal parameters in all participants and the male participant cases. A significant difference was also exhibited at the slower, preferred, and faster speeds in the GV parameters, except the CV of the double support phase (all and male: preferred vs. faster) and GA (all: preferred vs. faster; male: slower vs. preferred/ preferred vs. faster). For the high TFMS group, all spatiotemporal and GV parameters were significantly different at the slower, preferred, and faster speeds for all participants and the male participant cases (Tables 2 and 3).

For female participants, all spatiotemporal parameters showed a significant difference at the slower, preferred, and faster speeds, and the GV parameters were also significantly different among all speeds, except the CV of the double support phase and the GA between the preferred and faster speed conditions for the low and high TFMS groups (Table 4).

### Classifier variables for the low and high TFMS groups

The stepwise binary logistic regression analysis for the low TFMS and high TFMS groups revealed that the CV of the stride length at faster speed was significantly different in all participants (odds ratio [OR]: 1.985, 95% confidence interval [CI]: 1.290–3.056, *p* = 0.002). For male participants, the CV of the double support phase at faster speed was significantly different between the two groups (OR: 1.208, 95% CI [1.040–1.405], *p* = 0.014). For female participants, the CV of the stride length at slower speed was significantly different between the two groups (OR: 1.483, 95% CI [1.068–2.059], *p* = 0.019) (Table 5).

**Table 5:** Binary logistic regression results for the low TFMS and high TFMS groups.

**Variables**	**Estimate**	**SE**	**OR**	**95% CI for the OR**	***p*-value**
**All participants**					
CV of stride length at faster speed	0.686	0.220	1.985	1.290–3.056	0.002
**Male participants**					
CV of double support phase at faster speed	0.189	0.077	1.208	1.040–1.405	0.014
**Female participants**					
CV of stride time at slower speed	0.394	0.167	1.483	1.068–2.059	0.019

**Notes.**

Model adjusted for BMI and % body fat.

CI confidence interval OR odds ratio SE standard error

### Relationship between the TFMS and functional characteristics for the PA and stress responses of all participants

The TFMS showed a positive correlation with vigorous PA (all participants: *r* = 0.099, *p* < 0.05; women: *r* = 0.164, *p* < 0.05), moderate PA (all participant: *r* = 0.179, *p* < 0.05; male: *r* = 0.161, *p* < 0.05; female: *r* = 0.194, *p* < 0.05), and total PA (all participants: *r* = 0.125, *p* < 0.05). In addition, the TFMS was negatively correlated to body weight (female: *r* = −0.156, *p* < 0.05), BMI (all participants: *r* = −0.095, *p* < 0.05; female: *r* = −0.142, *p* < 0.05), percent body fat (all participants: *r* = −0.190, *p* < 0.05; male: *r* = −0.205, *p* < 0.05; female: *r* = −0.205, *p* < 0.05), and SRI-MF (all participants: *r* = −0.181, *p* < 0.05; male: *r* = −0.243, *p* < 0.05) (Table 6).

**Table 6:** Results of the correlation analysis between the TFMS and demographic characteristics.

**Variables**	All **Participants** *N* = 439	**Male** **Participants** *N* = 203	**Female** Participants *N* = 236
Age (years)	−0.003	−0.038	0.010
Height (cm)	−0.010	−0.091	−0.064
Body weight (kg)	−0.073	−0.105	−0.156^*^
BMI (kg/m^2^)	−0.095^*^	−0.080	−0.142^*^
Percent body fat (%)	-0.190^*^	−0.205^*^	−0.225^*^
SRI-MF (score)	−0.181^*^	−0.243^*^	−0.111
Vigorous PA (METs)	0.099^*^	0.037	0.164^*^
Moderate PA (METs)	0.179^*^	0.161^*^	0.194^*^
Walking PA (METs)	0.033	0.112	−0.026
Total PA (METs)	0.125^*^	0.115	0.120

**Notes.**

BMI Body mass index TFMS Total Functional Movement screen score SRI-MF Stress response index modified from PA Physical activity

* *p* < 0.05.

### Relationship between the TFMS and gait variables of all participants

For all participants, the TFMS showed a positive correlation with the walking speed, stride length, and step length at all speed conditions. The TFMS showed a negative correlation with the GV parameters (slower: CV of stride length; preferred: CVs of stride length, single support phase, stance phase, stride time, and step time; faster: all GV parameters) and bilateral coordination (slower and preferred: GA and PCI; faster: PCI). For male participants, TFMS showed a positive correlation with the stride length and step length at all speed conditions. However, a negative correlation was observed with all GV parameters at the faster speed condition and bilateral coordination (slower: PCI; faster: GA). For female participants, TFMS showed a positive correlation with the single support phase at faster speed. In addition, a negative correlation was observed with the GV parameters (slower: CVs of stride length, step length, stance phase, stride time, and step time; preferred: CVs of stride length, single support phase, stance phase, and stride time; faster: CVs of stride length, step length, single support phase, double support phase, stride time, and step time) and bilateral coordination (slower: PCI; preferred: GA) (Table 7).

**Table 7:** Results of the correlation analysis between the TFMS and gait variables.

Variables	All **Participants** *N* = 439
		**Male** **Participants** *N* = 203	**Female** **Participants** *N* = 236
**Slower speed**			
Walking speed (m/s)	0.107^*^	0.102	0.094
Stride length (m)	0.141^*^	0.163^*^	0.105
Step length (m)	0.140^*^	0.153^*^	0.111
GA (%)	−0.109^*^	−0.095	−0.125
PCI (%)	−0.171^*^	−0.203^*^	−0.134^*^
CV of stride length (%)	−0.097^*^	0.021	−0.196^*^
CV of step length (%)	−0.063	0.003	−0.135^*^
CV of stance phase (%)	−0.097*	−0.039	−0.157^*^
CV of stride time (%)	−0.079	0.021	−0.196^*^
CV of step time (%)	−0.067	0.005	−0.150^*^
**Preferred Speed**			
Walking speed (m/s)	0.105^*^	0.097	0.094
Stride length (m)	0.131^*^	0.170^*^	0.079
Step length (m)	0.132^*^	0.171^*^	0.081
GA (%)	−0.110^*^	−0.091	−0.133^*^
PCI (%)	−0.121^*^	−0.117	−0.116
CV of stride length (%)	−0.107^*^	−0.049	−0.151^*^
CV of single support phase (%)	−0.098^*^	−0.045	−0.128^*^
CV of stance phase (%)	−0.120^*^	−0.082	−0.148^*^
CV of stride time (%)	−0.107^*^	−0.049	−0.151^*^
CV of step time (%)	−0.098^*^	−0.064	−0.119
**Faster speed**			
Walking speed (m/s)	0.104^*^	0.088	0.099
Stride length (m)	0.138^*^	0.154^*^	0.107
Step length (m)	0.137^*^	0.151^*^	0.107
Single support phase (%)	0.066	−0.017	0.130^*^
GA (%)	-0.114^*^	−0.145^*^	−0.082
CV of stride length (%)	−0.190^*^	−0.190^*^	−0.185^*^
CV of step length (%)	−0.164^*^	−0.163^*^	−0.160^*^
CV of single support phase (%)	−0.149^*^	−0.161^*^	−0.131^*^
CV of double support phase (%)	−0.112^*^	−0.217^*^	−0.011
CV of stance phase (%)	−0.176^*^	−0.169^*^	−0.177
CV of stride time (%)	−0.190^*^	−0.190^*^	−0.185^*^
CV of step time (%)	−0.170^*^	−0.183^*^	−0.153^*^

**Notes.**

CV Coefficient of variance GA Gait asymmetry PCI Phase coordinate index TFMS Total Functional Movement screen score

* *p* < 0.05.

## Discussion

The main findings of this study are as follows: (1) The low TFMS group exhibited a shortened stride and step length (in all speed conditions) and worsen values of GV (faster) and bilateral coordination (slower) than the high TFMS group for all participants and in the male participant case. The female participants in the low TFMS group showed worsen GV values (slower and faster) than those in the high TFMS group. (2) The GV parameter was a crucial variable for distinguishing the low TFMS and high TFMS groups (all: CV of stride length at faster speed; male: CV of double support phase at faster speed; and female: CV of stride time at slower speed). (3) The low TFMS group demonstrated insufficient gait adaptations between the preferred and faster speeds, as indicated by the CV of the double support phase and GA. (4) A lower TFMS was associated with declined gait ability, as indicated by the increased GV parameters and bilateral coordination. These findings are discussed in detail below.

A low TFMS may indicate movement deficits (Cook et al., 2014), which may contribute to poor fundamental movement patterns. Thus, we hypothesized that the participants in the low TFMS group would exhibit poor gait ability, and this hypothesis was verified. The low TFMS group exhibited slower walking speeds and greater values of GV and PCI, which reflects worse gait stability than the high TFMS group for all participants and sexes. A slower walking speed and shortened stride length reflect weakness of the lower limbs (Herssens et al., 2018; Aboutorabi et al., 2016). Because these patterns may lead to longer double support and stance phases and a shorter single support phase (Perry & Davids, 1992), more attention and dynamic stability during walking may be required owing to the increasing time spent in the single-limb stance and mediolateral displacement of the center of mass (Rennie et al., 2018; Nasirzade et al., 2017). These gait patterns may also contribute to an increase in the GV and coordination of the left and right limbs during walking (Plotnik, Giladi & Hausdorff, 2007; Nasirzade et al., 2017; Plotnik et al., 2013). Greater values of GV and bilateral coordination reflect poor gait stability due to a decline in muscle strength and motor function (Herssens et al., 2018; Almarwani et al., 2016; Plotnik, Giladi & Hausdorff, 2007; Plotnik et al., 2013). We did not measure the muscle strength; however, the possibility that the low TFMS group exhibited less moderate PA than the high TFMS group was demonstrated for all participants and the male participant cases. Furthermore, the TFMS was correlated with the physical characteristics (BMI and % body fat), stress index, and PA levels for all participants, and these results were similar with previous studies that the TFMS was negatively correlated with BMI in the general population (non-athletes or military) (Perry & Koehle, 2013; Koehle et al., 2016). Perry & Koehle, (2013) demonstrated that participants with BMI ≥ 30 exhibited lower TFMS compared to participants with BMI <30, and they reported that obesity limits human movement and increases musculoskeletal or joint-related pain during movement. They also reported higher PA levels associated with higher TFMS. However, they recognized that even though the BMI and PA levels were associated with the TFMS, other associated variables may also exist. Our study reported that stress level (SRI-MF) was associated with the TFMS, and the low TFMS group exhibited higher stress levels than the high TFMS group in all participants and male cases, which means that a severe stress level may negatively affect the fundamental movement functions. In summary, our results demonstrated that young adults with a TFMS ≤ 14 may have low-quality physical characteristics and severe stress level. Consequently, the low TFMS group exhibited reduced gait ability compared to the high TFMS group.

Our logistic analysis results to classify the low TFMS and high TFMS groups indicated different gait characteristics according to sex. For all participants, CV of the stride length at a faster speed was approximately 98.5% greater for the low TFMS group than for the high TFMS group. Similar results were also observed for the male participants. The CV of the double support phase at a faster speed was approximately 20.8% greater in the low TFMS than in the high TFMS group. These results reflect that the walking task at the faster speed condition may be challenging because it requires more mechanical energy and thus increased muscle activity (Neptune, Sasaki & Kautz, 2008). In addition, the male participants in the low TFMS group demonstrated reduced gait stability with lower PA levels, which may be related to muscle weakness due to low PA levels (Oh-Park et al., 2011; Pau et al., 2014). Conversely, for the female participants in the low TFMS group, the CV of the stride time at a slower speed was approximately 48.3% greater than that in women of the high TFMS group. The walking task at a slower speed may also be challenging because a reduced gait automaticity was indicated even in healthy young adults because of the higher cortical control requirement with the change in muscle activation patterns from the preferred speeds (Almarwani et al., 2016). Thus, a decline in the central motor control and the automatic stepping mechanism during walking is possible (Herssens et al., 2018; Almarwani et al., 2016), and the results obtained for females in the low TFMS group may be related to these symptoms.

As the speed gradually increased from slower to faster, the high TFMS group for all participants and for the male participant cases exhibited significant differences for all gait variables. An increasing trend in the walking speed, stride length, step length, and single support phase was demonstrated. There was a significant decreasing trend in the double support phase, stance phase, all CVs of the spatiotemporal parameters, and bilateral coordination. The low TFMS group for all participants, irrespective of sex, also exhibited significant differences in most gait variables; however, no significant differences in the CV of the double support phase (all participants and sexes: preferred vs. faster) and GA (all participants and the female cases: preferred vs. faster; male case: slower vs. preferred and preferred vs. faster) were observed. These patterns may be one of the symptoms of declining gait stability in the low TFMS group. The GV value is decreased with increasing walking speed due to enhanced dynamic stability during walking (Lee et al., 2020c). In addition, control of the walking-related rhythmic stepping mechanism is reflected by stride-to-stride variability (Gabell & Nayak, 1984), and the lower variability reflects automatic processes that require minimal attention, which can be attributed to efficient gait control and stability (Hausdorff et al., 2005). Similar results that conducting the treadmill walking test with slower, preferred, and faster speed conditions reported the control group (healthy elderly female) exhibited significant increasing and decreasing trends for all gait variables, whereas the experimental group (sustained subthreshold insomnia stage) exhibited no significant differences in the CVs of stride length, stride time, and step time between the slower and preferred speeds. They suggested these patterns may be related to the symptoms of declining gait stability by reduced gait adaptation ability (Lee et al., 2020a). However, the high TFMS group in the female case showed no significant differences as in the all participant and the male participant cases in the CV of the double support phase and GA variables (preferred vs. faster). This may be because the PA and stress levels of the high TFMS group for the female case were not significantly different from those of the low TFMS group which may be due to the fact that the female participants exhibited relatively lower PA and higher stress levels regardless of the TFMS.

In general, the TFMS showed a positive correlation with walking speed, stride length, and step length (range of *r*: 0.105–0.171) and a negative correlation with the GV and bilateral coordination (range of *r*: −0.098 to −0.217). In particular, the CVs of stride length (*r*: −0.190) and double support phase (*r*: −0.217) at the faster speed condition and the CV of stride time at the slower speed (*r*: −0.196) condition indicated the highest correlation results among all significant variables, which were also the classifiers between the low TFMS and high TFMS groups for all participants and for the male participant and female participant cases. These results support the fact that poor functional movement patterns are associated with a decline of gait ability. We demonstrated differences in the association between TFMS and gait ability according to sex. The poor GV outcomes may indicate a decline in gait performance because worsened gait performance may cause motor function decline, which is related to the control of gait patterns (Herssens et al., 2018). Even though our participants were not athletes, those with a TFMS ≤ 14 could be exposed to potential musculoskeletal injury risks or sustained decreased fundamental movement function in daily life. Therefore, the participants with a lower TFMS (under 14) and poor gait ability may need to enroll in intervention programs to prevent future risk of injury and enhance motor function.

We analyzed the association between the fundamental movement function and gait ability in young adults, and our results indicated that participants with a poor fundamental movement function exhibited decreased gait ability during walking tasks at slower and faster speed conditions. However, there are limitations associated with this study. First, we considered TFMS to be a composite score using seven fundamental movement patterns scores. However, some studies have reported that the TFMS is not effective for identifying future risks of injury in athletes (Moran et al., 2017; Trinidad-Fernandez, Gonzalez-Sanchez & Cuesta-Vargas, 2019; Moore et al., 2019). Specific characteristics may be associated with risk of injury including participant characteristics, such as age, sex, sports, and injury types (Moran et al., 2017). Li et al. (2015) suggested considering FMS sub-score rather than TFMS to evaluate movement function. Thus, future studies should consider the essential characteristics of FMS, such as compensation movement patterns or specific pain occurrences, which may be meaningful to understand the gait characteristics according to the FMS characteristics. In addition, we did not measure the muscle strength using objective measurements (e.g., hand grip, peak torque). Thus, future studies should consider measuring the muscular strength which may provide insights into the gait characteristics, according to the FMS performances.

## Conclusions

The low TFMS group exhibited reduced gait ability compared to the high TFMS group. The different gait characteristics between the low TFMS and high TFMS groups were indicated based on sex. Male participants in the low TFMS group indicated a greater CV of the double support phase at a faster speed, which may be related to muscle strength weakness. Female participants in the low TFMS group indicated a greater CV of stride time at a slower speed, which may be related to a decline in central motor control during walking. Hence, the low TFMS group demonstrated insufficient gait adaptations at preferred and faster speeds compared to the high TFMS group, as indicated by the CV of the double support phase and GA. Finally, the poor functional movement patterns were found to be associated with a decline in gait ability. Therefore, gait tasks at various speed conditions are useful for evaluating the fundamental movement functions in healthy young adults who are aged between 19 and 30 years. Participants with a lower TFMS and poor gait ability may require intervention programs to prevent risk of future injury and enhance motor functions.

##  Supplemental Information

10.7717/peerj.11356/supp-1 File S1 Raw data showing all parameters

## Additional Information and Declarations

### Data Availability

The following information was supplied regarding data availability: The raw measurements are available in the Supplemental File.
